# Visually estimating body mass of wild pigs

**DOI:** 10.1038/s41598-025-22857-8

**Published:** 2025-11-27

**Authors:** Nathan P. Snow, Kelly J. Koriakin, Michael J. Lavelle, Michael P. Glow, Justin W. Fischer, Justin A. Foster, Kurt C. VerCauteren

**Affiliations:** 1 United States Department of Agriculture, Animal and Plant Health Inspection Service, Wildlife Services, National Wildlife Research Center, Fort Collins, CO USA; 2 Texas Parks and Wildlife Department, Kerr Wildlife Management Area, Hunt, TX USA

**Keywords:** Body condition, Body mass, Feral swine, Morphology, Sus scrofa, Wild boar, Weight, Wildlife damage management, Invasive species, Population dynamics

## Abstract

Body mass of wild pigs (*Sus scrofa*) can provide useful information regarding reproductive capacity of a population, and population health and resilience for this highly-destructive invasive species. Body mass of females is an indicator of whether they reproduce before 1 year of age, which could have substantial impacts on reproductive capacity of a population. Measuring body mass can be difficult because large wild pigs may require > 1 person to weigh, are often located in remote areas making equipment difficult to transport, or are often culled without access to the carcass (e.g., shooting from aircraft). We evaluated our ability to accurately estimate the body mass of wild pigs by visual inspection, and identified which factors (i.e., morphometrics and demographics) influenced the accuracy of our estimates. We visually estimated the body mass of wild pigs and then collected actual weights of 1,210 wild pigs across 5 regions (Alabama, Texas, Hawaii, Guam, Queensland). We also collected morphometric measurements and age to evaluate how these factors impacted our estimates. On average we found our estimates were accurate, averaging only -0.14 kg underestimated weights across all wild pigs weighed. However, our estimates were most severely underestimated (e.g., up to -20 kg) for younger wild pigs (i.e., < 1–3 years) that were heavier (i.e., > 30 kg). We also confirmed that although growth rates slowed after 1 year of age, wild pigs continued to grow in body length, head length, height, and girth as they aged, which explained why the age of an animal influenced our ability to generate accurate estimates. We surmised that young-yet-heavy wild pigs were disproportionally stouter than older animals, thus were underestimated due to their shortened appearance. Underestimating the body mass for young-yet-heavy females could misinform management plans, because these animals may have substantial influence on the reproductive capacity of a population. For visually estimating body mass of wild pigs, we recommend considering indicators of age (e.g., morphological proportions) to avoid underestimating young-yet-heavy animals with stout appearances. We also recommend calibrating observers regularly using known weights and morphometrics.

## Introduction

Wild pigs (*Sus scrofa;* feral domestic swine, wild boar, or hybrids between these two parental forms) are a destructive invasive species^1^ that are established across many regions of the world, including North and South America, Australia, and many island nations^2–6^. Wild pigs are considered pests throughout much of their established range^7,8^ because of damage they cause to agriculture, natural resources, and property^3,9,10^. Wild pigs also pose threats to humans and livestock as vectors of disease and causes of injury^11–13^. Accordingly, wild pigs were listed in the top 100 worst invasive alien species on Earth^14^.

Management of wild pigs using population reduction is common in locations where they cause large amounts of damage to agriculture and natural resources^15^. During lethal removal, it is useful to determine the sizes (e.g., body mass) of the wild pigs to understand the impact of the management effort^16,17^. For example, collecting body mass measurements is helpful for understanding the relationship between productivity and resource availability, reproductive potential, human health risks, age class, and body condition^18^. Specifically, female wild pigs that reach ≥ 30 kg within their first year are more likely to reproduce during that same year, which indicates a highly productive population^16^. Further, comparing trends of body mass through time and regions can provide valuable insights on the health and condition of wild pigs^16,17^. Despite the importance of this measurement, accurately assessing body mass can be challenging because wild pigs are large and difficult to lift, and remoteness of where they live can make transporting weighing equipment difficult if not impossible^17^.

In the absence of weighing wild pigs, equations have been developed that utilize morphometric measurements (e.g., body length or chest girth) to estimate the body mass of wild pigs^17,18^. However, there are situations when obtaining morphometric measurements are not possible. For example, when culling wild pigs using aircraft, when loading live-trapped wild pigs into a transport trailer (e.g., and not immediately culling), or when attempting to chemical immobilize live-trapped wild pigs for research purposes. In these situations, wildlife professionals estimate the weights of wild pigs using their previous experience with the animals, often with unknown accuracy.

Our objective was to evaluate how accurate our estimates of body weights were for wild pigs and to determine factors that might have influenced that accuracy. We opportunistically estimated weights of wild pigs that we captured and marked, or culled, as parts of various research projects through the USA and Australia during 2016–2024. We compared our estimates to actual body mass weights and examined if our estimates were biased based on sizes and age of wild pigs (body mass and morphometric measurements), and our experience (i.e., duration of the project). Our overall goal was to provide information on which factor(s) impacted discrepancies between estimated and actual weights, and how to improve accuracy.

## Methods and materials

### Study area

**Fig. 1:**
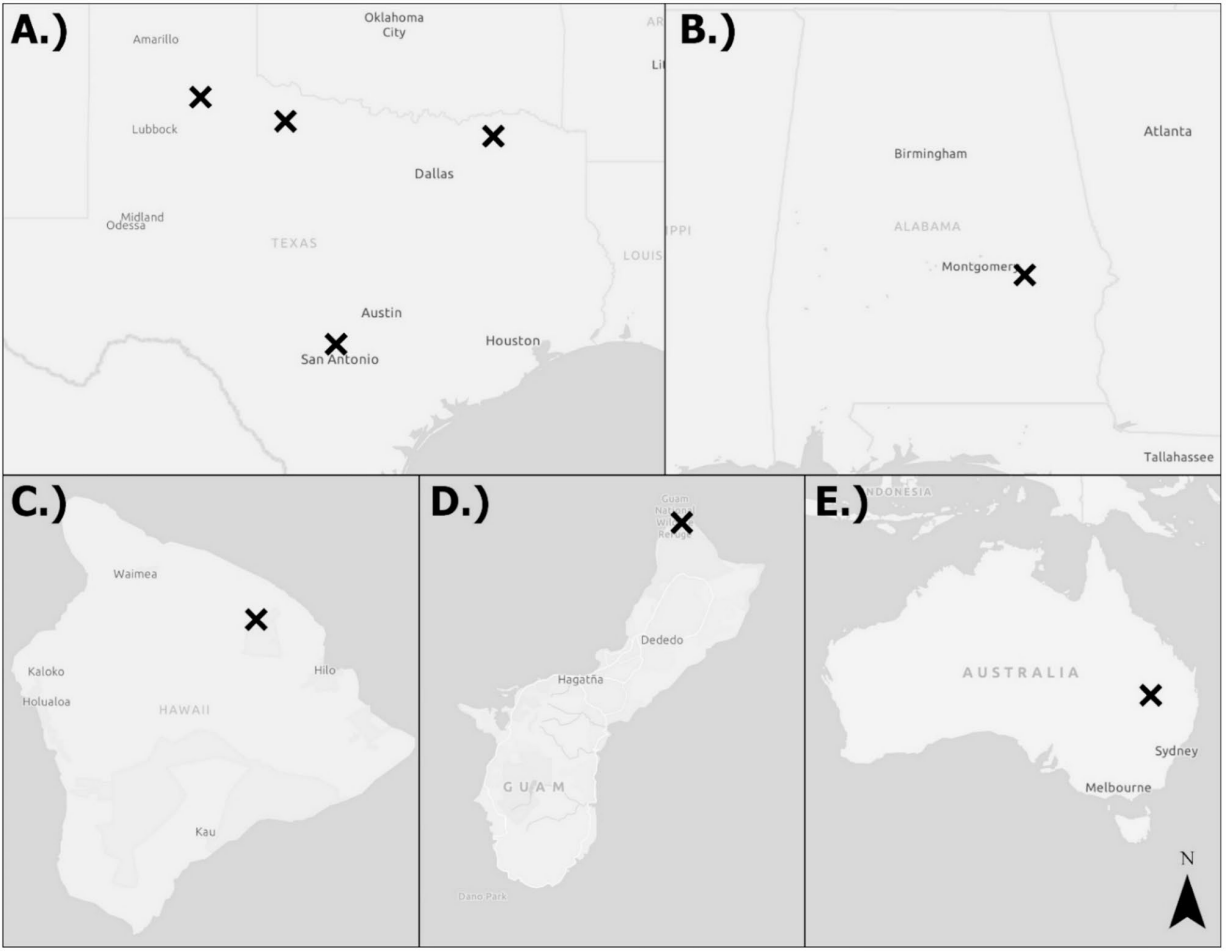
Study sites where wild pigs (*Sus scrofa*) were sampled throughout the world during 2016–2024. (**A**) Texas, USA; (**B**) Alabama, USA; (**C**) Hawai’i, USA, (**D**) Guam, USA, (**E**) Queensland, AU. Map generated using ArcGIS Pro (Esri, Redlands California, USA).

We opportunistically sampled wild pig populations from study sites spread across five regions (Fig. 1) during 2016–2024 described in detail in Snow, et al. ^16^. We grouped the regions as two sites from the continental United States (i.e., Alabama and Texas), two sites from islands of the United States (i.e., Guam and Hawai’i), and one site from continental Australia (i.e., Queensland). In brief, the potential population densities of wild pigs ranged from an estimated ~ 3–11 wild pigs/km^2^ among the regions^19^. Alabama was characterized by a mixture of croplands, pasturelands, and woodland forests, with average temperatures from 13 to 17 °C and annual precipitation at 1,229 mm. Texas was a mosaic of cropland, rangeland, grasslands, and woodlands, with average annual temperature from 10 to 21 °C and precipitation from 433 to 954 mm. The study area on Guam was primarily limestone forests with patches of scrub/shrub and grasslands, with average temperatures of 27.8 °C and rainfall of 2,133–2,946 mm. The study area on Hawai’i was characterized by a mix of montane forest with open and closed canopy and grasslands, with annual temperatures averaging 15 °C and annual precipitation of 2,500 mm. Finally, the study area in Queensland was a temperate grassland, savanna, and shrubland ecoregion with undulating plains and low hills, with temperatures averaging 21 °C and precipitation totals of 305 mm.

### Data collection

We collected data from 2,762 wild pigs across the 5 regions: including 156 in Alabama, 1,943 in Texas, 110 in Guam, 490 in Hawai’i, and 63 in Queensland. As described in Snow, et al. ^16^, we sampled wild pigs killed or immobilized during research and operational control activities, including testing a toxic bait studies: ^20–25^, population control using aerial operations studies:^20,24^, and eradication attempts with trapping and shooting studies: ^26–28^. We generally targeted groups of wild pigs but opportunistically sampled solo animals when possible. We did not target removing or sampling specific age classes or sexes of wild pigs during these activities. All data were collected using mobile devices with ArcGIS Survey123 digital forms (ESRI, Redlands, CA USA). All research methods were approved by the United States Department of Agriculture, National Wildlife Research Center Institutional Animal Care and Use Committee (protocols: QA-2612, QA-2724, QA-2990, QA-3068, QA-3225, QA-3311, QA-3312, QA-3407, and QA-3470), and performed and reported in accordance with Animal Research: Reporting of In Vivo Experiments (ARRIVE) guidelines^29^.

Data collectors were a mixture of experienced and inexperienced wildlife professionals, ranging from 0 to 15 + years of experience with wild pigs. Inexperienced folks were paired with a senior member of the team for training. Most of the senior members of the team were present during all years of data collection, whereas lessor experienced members were present for 1–3 years.

We collected similar data from dead or immobilized wild pigs throughout all activities. Specifically, the first record we collected was an estimate of weight based on a quick visual inspection of the animal. This estimate was done to the best of our ability and based on our previous experiences with handling wild pigs. Often this estimate was based on a consensus among a team of ≥ 2 observers. Then, we measured the actual body mass using hanging scales (e.g., HME-Scale, Hunting Made Easy, Irving, TX) or platform scales (e.g., W110 Weigh Scale System with 3300# Load Bars, Gallagher, Riverside, MO). We recorded estimates of body mass to the nearest 1.0 lb and actual weights to the nearest 0.1 lb (hanging scale) or 0.5 lb (platform scale), and converted all measurements to kg for the purposes of this analysis.

We calculated the cumulative day of each study to account for our previous experience with estimating weights of wild pigs. For example, we assumed our estimation accuracy increased as the days of data collection ensued because we were checking our estimates with actual weights. For example, we considered the first day of data collection for each data collection effort as “1”, the second day as “2”, and so on. The count of days was reset for each effort. As such, this variable was not relative to overall professional experience, instead was a measure of experience with observing new populations of wild pigs in new locations.

We estimated the age of wild pigs using a standardized aging guide^30^ that was developed from published aging standards^31–34^. The guide was supplemented with a stepwise key for consistent classification among researchers using photographs and diagrams. Specifically, we used several defining characteristics such as replacement of deciduous or eruption of permanent teeth and tooth wear to classify wild pigs into one of nine age classes described in detail in:^16^, consisting of 0–8 weeks, 8–20 weeks, 20–30 weeks, 30–51 weeks, 12–18 months, 18–26 months, 26–36 months, 36–48 months, and > 48 months. From each of these classes, we estimated the minimum and maximum possible age of each wild pig. We considered the minimum age for the 0–8 weeks age class to be 0.5 months (i.e., out of the birthing nest), and the maximum age for the > 48 months to be 96 months (i.e., the oldest that wild pigs reached)^35^. Then, from the minimum and maximum possible range for each age class, we calculated the median estimated age. We used these median estimated age as a continuous variable in subsequent analyses.

Following methods outlined in Baruzzi, et al. ^17^, we recorded morphometric measurements of wild pigs including: full body length (i.e., tip of nose to base of tail), height at shoulder, and girth at heart. We also recorded head length (i.e., tip of nose to nuchal crest). We recorded these measurements to the nearest 0.25 in, and converted to cm for this analysis. We also recorded the sex of wild pigs. Finally, we recorded if a female was actively reproductive at the time of sampling, if they were obviously pregnant (i.e., distended belly) or recently pregnant (i.e., actively lactating nipples). We were unable to collect every component of data from some wild pigs (e.g., scavenged carcasses were not weighed, but could be aged and sometimes sexed). Overall, we had 1,210 wild pigs in which all components of data described above were collected and these represented the final sample size for our analyses below. These samples included 97 wild pigs from Alabama, 625 from Texas, 413 from Hawaii, 40 from Guam, and 35 from Queensland.

### Data analysis

All analyses were conducted in Program R (v4.2.0; The R Foundation for Statistical Computing). We calculated the difference between our estimated and actual body mass (i.e., estimated – actual = discrepancy) and considered these values as the response variable. Our list of independent variables included: sex (F, M), age (months), actual weight (kg), body length (cm), head length (cm), girth (cm), height (cm), and day of study (day). We evaluated correlation among independent variables and excluded head length, girth, and height because they were highly correlated (r >= |0.6|) with body length. After initial evaluation, we log-transformed age because early growth of wild pigs was rapid and slowed as the animals got older. We also evaluated if log-transforming day of study improved model fit, assuming learning was rapid and slowed throughout the study, but fit was not improved therefore we used the non-transformed variable. We did not include region as a predictor because we wanted to make our inferences broadly applicable to all wild pigs. We also did not include observer as a predictor because often the estimates of body mass were based on consensus of ≥ 2 observers.

We used a linear model to examine how the remaining variables influenced our ability to accurately estimate the weights of wild pigs. Specifically, we examined the global model: estimated weight discrepancy ~ actual weight + sex + age + body length + day of study + (actual weight × age) + (sex × age) + (sex × actual weight). We used the three interactions to evaluate different relationships among sex and age. We evaluated all combinations of the global model and calculated the relative importance, model-averaged parameter coefficients, and 95% confidence intervals for all variables in the top ranked models (∆AICc ≤ 2.0). We considered meaningful differences for any parameter estimates with lack of overlap of zero from 95% confidence intervals (CIs), and made inferences about the biological significance by plotting model predictions with 95% prediction intervals (PIs).

*Post hoc*, we conducted another set of analyses to examine how the morphometric body measurements of wild pigs changed as the animals aged. Specifically, we conducted a set of four univariate linear mixed models to examine how the responses of body length, head length, height, and girth, respectively, changed as a function of ~ sex + age + (sex × age). We also considered the region that the animals were sampled from (i.e., Alabama, Texas, Hawaii, Guam, and Queensland) as a random intercept to account for regional variation that occurred^16^. We plotted the model predictions and 95% PIs for each body measurement.

We conducted one additional *post hoc* analysis to determine whether the reproductive status of young female wild pigs influenced our ability to accurately estimate their body mass. Specifically, we evaluated whether young females that were obviously pregnant or recently pregnant influenced our estimates. For this analysis, we trimmed our data to only include only females that were 6–18 months old. We evaluated the linear mixed model of: discrepancy ~ age + reproductive status + (age × reproductive status). Reproductive status was a yes/no for whether the female was actively reproductive at the time of sampling. We also included a random intercept for region as described above.

## Results

**Fig. 2:**
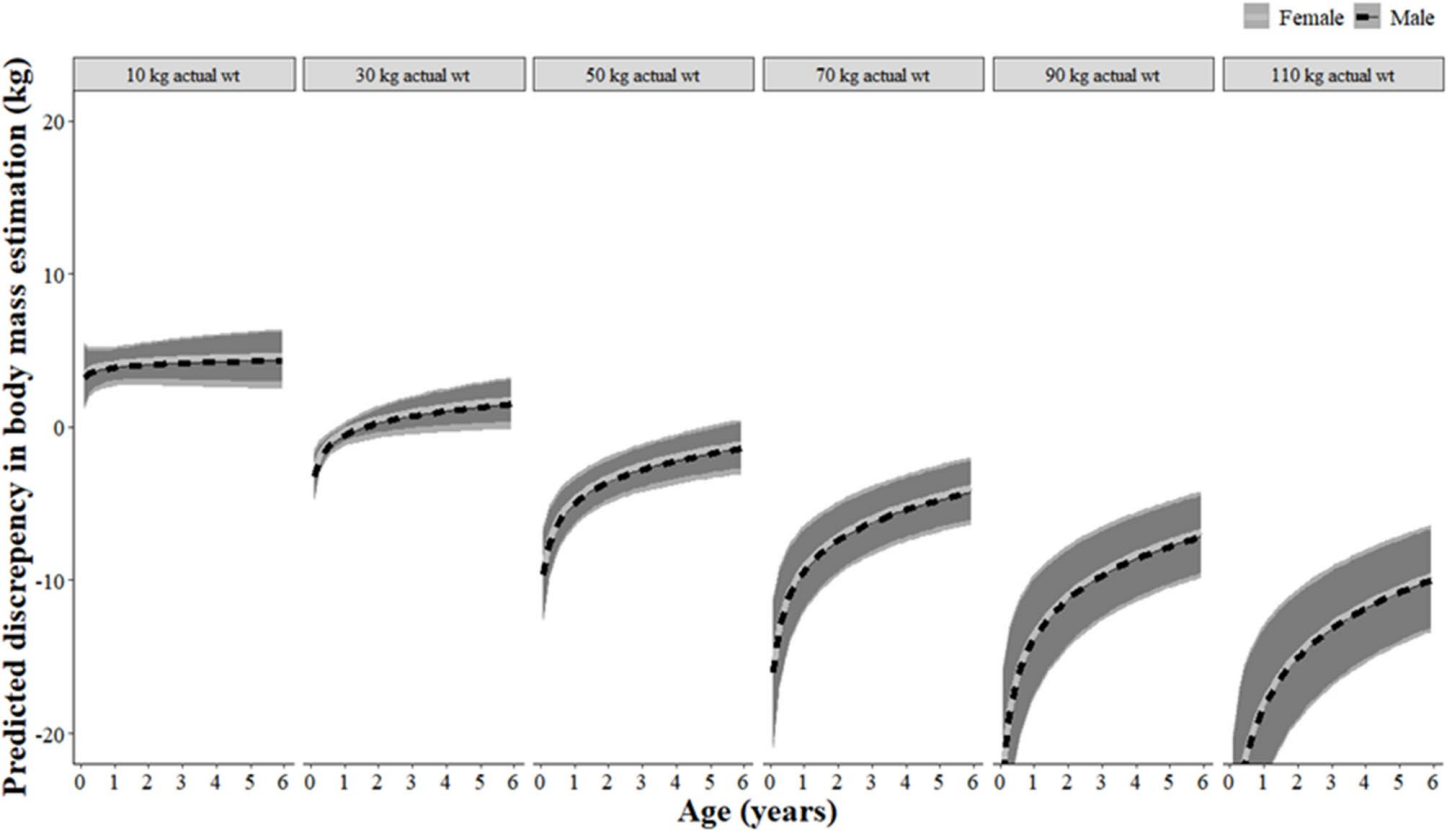
Model predicted discrepancies (kg) in visually estimated body mass (and 95% prediction intervals), depicted by body mass categories (held constant at: 10, 30, 50, 70, 90, and 110 kg) for wild pigs (*Sus scrofa*) from five regions (Alabama, USA; Texas, USA; Hawaii, USA; Guam, USA; Queensland, AU) during 2016–2024.

**Fig. 3:**
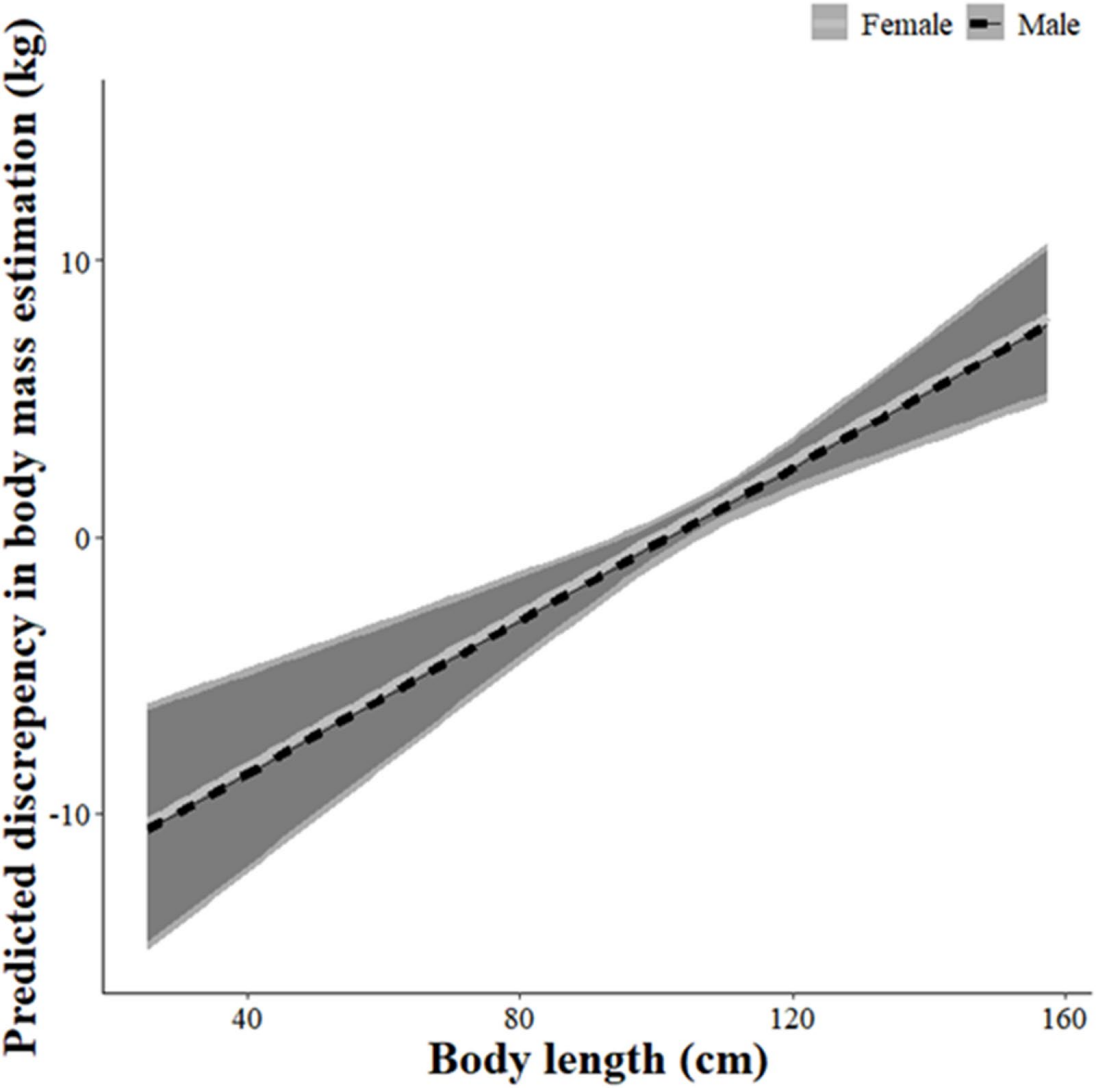
Model predicted discrepancies (kg) in visually estimated body mass (and 95% prediction intervals) by body length (i.e., cm; tip of nose to base of tail) for wild pigs (*Sus scrofa*) from five regions (Alabama, USA; Texas, USA; Hawaii, USA; Guam, USA; Queensland, AU) during 2016–2024.

From the 1,210 wild pigs, the largest actual weight observed was a 116.6 kg female that was lactating, and the smallest was a 0.7 kg female piglet. The discrepancy in our estimated body mass compared to the actual weights averaged -0.14 kg (SE = 0.17) but ranged from -48.31 to 40.64 kg. From model ranking, we found the top models included 5–7 covariates: actual weight, age, actual weight × age, body length, sex, day of study, and (age × sex) (Table 1). Actual weight, age, (actual weight × age), and body length all had relative importance values of 1.00, indicating these variables were the most influential for accurately estimating weights (Table 2). The interaction of (actual weight × age) substantially influenced our ability to accurately estimate the weights of wild pigs, where our estimates were most severely underestimated (e.g., up to -20 kg) for younger wild pigs (i.e., < 1–3 years) that were heavier (i.e., > 30 kg; Fig. 2). Additionally, body length influenced our ability to estimate weights, where we tended to underestimate (e.g., up to -10 kg) for shorter animals and overestimate (e.g., up to 8 kg) for longer animals (Fig. 3).

**Table 1:** Top models from model selection for predicting discrepancies in visually estimated body mass of wild pigs (*Sus scrofa*) from five regions (Alabama, USA; Texas, USA; Hawaii, USA; Guam, USA; Queensland, AU) during 2016–2024.

Model	No. parameters^a^	∆AICc	Model weight
Actual weight + age + actual weight × age + body length + sex	7		0.29
Actual weight + age + actual weight × age + body length	6	0.08	0.28
Actual weight + age + actual weight × age + body length + day of study	7	1.17	0.16
Actual weight + age + actual weight × age + body length + day of study + sex	8	1.28	0.15
Actual weight + age + actual weight × age + body length + sex + age × sex	8	1.80	0.12

^a^Number of parameters including an intercept.

**Table 2:** Model-averaged parameter results from set of top-models for predicting discrepancies in visually estimated body mass of wild pigs (*Sus scrofa*) from five regions (Alabama, USA; Texas, USA; Hawaii, USA; Guam, USA; Queensland, AU) during 2016–2024.

Predictor	Relative importance	Parameter estimate	95% confidence interval	
			Lower	Upper
Actual weight	1.00	− 0.33	− 0.43	− 0.22
Age	1.00	− 0.15	− 1.00	0.71
Body length	1.00	0.14	0.09	0.19
Sex	1.00	− 0.23	− 1.29	0.45
Actual weight × age	0.56	0.04	0.02	0.06
Day of study	0.31	0.00	− 0.01	0.03
Age × sex	0.12	− 0.02	− 0.68	0.41

**Fig. 4:**
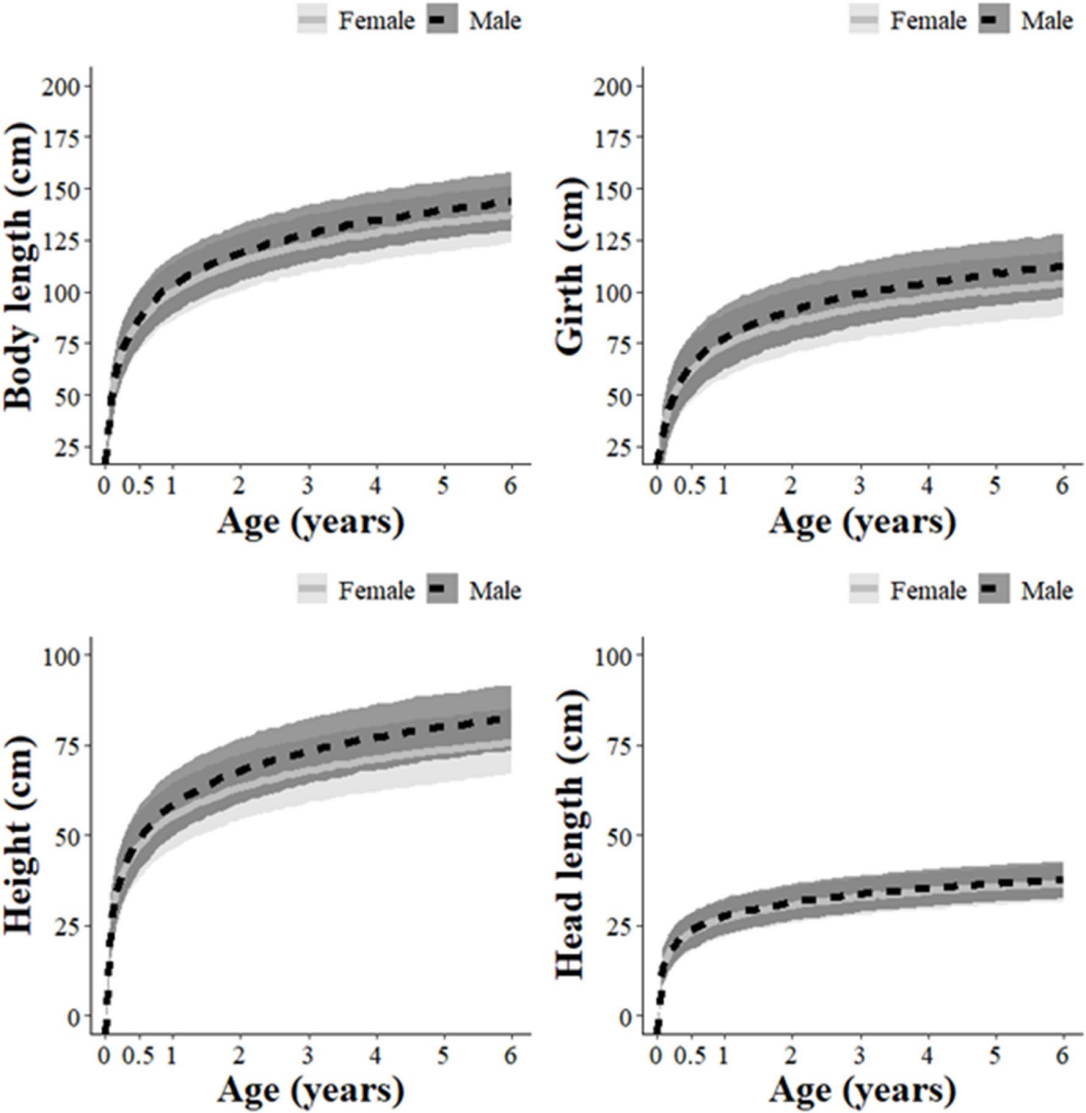
Predicted body length (cm; tip of nose to base of tail), girth (cm; at heart), height (cm; at shoulder), and head length (cm; tip of nose to nuchal crest) growth rates (with 95% CIs) by age (years) for wild pigs (*Sus scrofa*) from five regions (Alabama, USA; Texas, USA; Hawaii, USA; Guam, USA; Queensland, AU) during 2016–2024.

From the *post hoc* analysis, we showed that growth rates were similar between the sexes and confirmed that body length, head length, height, and girth continued increase as wild pigs aged. However, all measures followed logarithmic growth, where the rate of growth slowed as the animals aged (Fig. 4). We also found no indication that our body mass estimates were influenced by the interaction of (age × reproductive status) (β = -8.54; 95% CI = -22.61–5.52) or that main effect of reproductive status (β = 24.80; 95% CI = -12.30–61.91) for young, female wild pigs.

## Discussion

Visually estimating body mass of wild pigs was accurate (average = -0.14 kg) and reasonable for generalizing trends of population condition. However, we found some inaccuracies for groups of wild pigs that could impact this generalization. Our estimates were most severely underestimated (e.g., up to -20 kg) for younger wild pigs (i.e., < 1–3 years) that were heavier (i.e., > 30 kg). For females in particular, Snow, et al. ^16^ hypothesized that these young-yet-heavy animals were among the most influential to the population dynamics of wild pigs. If they reach ≥ 30 kg in their first year, they were more likely to reproduce within that first year and may have greater reproductive output^16^. Underestimating the weights of these young-yet-heavy animals could misinform the reproductive capacity of a population and misinform management plans, especially considering that reproduction by young-yet-heavy wild pigs could be a mechanism of compensatory reproduction^16,36^.

We conducted the *post hoc* analyses to understand why we underestimated body mass for young-yet-heavy wild pigs. We confirmed that wild pigs continued to grow in body length, head length, height, and girth as they aged (at least until ≥ 4–6 years old), similar to previous descriptions^37,38^. Considering this, we surmised that despite these young-yet-heavy animals reaching similar body mass as older wild pigs, they likely had disproportionally shorter morphometric measurements giving them a stout appearance. We likely underestimated them due to that shortened appearance. In addition, younger wild pigs have an immature hair morphology i.e., lack of underfur; ^37^ and smaller tusks, that could further bias observers toward lower estimates. Overall, we expect these indicators of age (i.e., body dimensions, hair morphology, tusk size) should be considered for informing estimates of body mass. Reproductive status of young females did not influence our estimates, indicating pregnancy of younger females did not complicate our estimation.

To further corroborate the above interpretations, we also found that the accuracy of estimates was dependent on body length. We underestimated body mass for wild pigs with shorter lengths and overestimated for longer lengths. The body length of wild pigs varies based on the age of the animal, but also because of variation in the total number of vertebrae ~ 50–55 vertebrae; ^37^. This variation is generated by natural variability, but also ancestry and introgression between different introduced sources of feral domestic swine, Eurasian wild boar, and their hybrids^39^, thus may vary by region. As such, it is important to regularly check estimates with actual weights to help observers calibrate their estimates, especially when working across regions.

We recommend exploration of how differing ancestries across regions might influence the morphology of wild pigs and subsequently the ability to accurately estimate body mass. For example in the US, multiple introductions of various breeds of domestic pigs and Eurasian wild boar occurred^40^, resulting in populations with mixed ancestries^41^ and likely unique morphometrics. Areas with high prevalence of Vietnamese pot-bellied pig genetics may tend to look stouter, for instance. A better understanding of how these differences might impact abilities to estimate weights would be a valuable line of research that could enhance regional estimates.

We recognize a few other limitations when interpreting these results. Our research teams were a mixture of experienced and inexperienced wildlife professionals, but we did not account for personal experience. We expected that personal experience was important, therefore lesser experienced individuals received training during each study to minimize this bias. Anecdotally, we observed that checking actual weights after making an estimate was very instructional, and subsequent estimates improved quickly (i.e., on the very next wild pig) for inexperienced folks. This may explain why day of study was not an important predictor, because observers were getting real time feedback from each animal and learning was happening quicker than a daily scale. Regardless, we caution readers from using estimated body mass when needing to make important management decisions. If accurate body mass are needed, we recommend using a scale. Furthermore, we were inherently checking our estimates for each wild pig by measuring the actual weight as part of our data collection, which may not be representative of population control efforts. Lastly, our estimates were made after a deliberate and close-proximity examination of the animal. We expect quicker visual observations and examination from farther distances (e.g., during aerial hunting) would have less accuracy.

## Management implications

For accurately estimating body mass of wild pigs, we recommend considering the potential age of the animals by examining morphology. Indicators like body length or hair morphology may reveal younger animals that tend to have body mass underestimated. Observers should try to avoid underestimating young-yet-heavy wild pigs with stout appearances because this can have implications on estimating population (or reproductive) productivity. We also recommend calibrating observers regularly using known weights and morphometrics.

## Data Availability

Data will be made available upon reasonable request to the corresponding author.

## Declarations

### Accordance

All data collection procedures were performed in accordance with relevant guidelines and regulations.

### Arrive

All procedures followed ARRIVE guidelines.
